# Ice‐Enabled Transfer of Graphene on Copper Substrates Enhanced by Electric Field and Cu_2_O

**DOI:** 10.1002/advs.202402319

**Published:** 2024-06-25

**Authors:** Hechuan Ma, Xiaoming Chen, Yufei Han, Jie Zhang, Kaiqiang Wen, Siyi Cheng, Quanyi Zhao, Yijie Wang, Jianyang Wu, Jinyou Shao

**Affiliations:** ^1^ Micro‐ and Nanotechnology Research Center State Key Laboratory for Manufacturing Systems Engineering Xi'an Jiaotong University Xi'an Shannxi 710049 China; ^2^ XJTU‐POLIMI Joint School of Design and Innovation Xi'an Jiaotong University Xi'an Shaanxi 710049 China; ^3^ Electronic Materials Research Lab Key Laboratory of the Ministry of Education Xi'an Jiaotong University Xi'an Shaanxi 710049 China; ^4^ Department of Physics Jiujiang Research Institute and Research Institute for Biomimetics and Soft Matter Fujian Provincial Key Laboratory for Soft Functional Materials Research Xiamen University Xiamen 361005 China

**Keywords:** electric field, graphene transfer, ice enabled transfer, interface control

## Abstract

Graphene films grown by the chemical vapor deposition (CVD) method suffer from contamination and damage during transfer. Herein, an innovative ice‐enabled transfer method under an applied electric field and in the presence of Cu_2_O (or Cu_2_O‐Electric‐field Ice Transfer, abbreviated as CEIT) is developed. Ice serves as a pollution‐free transfer medium while water molecules under the electric field fully wet the graphene surface for a bolstered adhesion force between the ice and graphene. Cu_2_O is used to reduce the adhesion force between graphene and copper. The combined methodology in CEIT ensures complete separation and clean transfer of graphene, resulting in successfully transferred graphene to various substrates, including polydimethylsiloxane (PDMS), Teflon, and C_4_F_8_ without pollution. The graphene obtained via CEIT is utilized to fabricate field‐effect transistors with electrical performances comparable to that of intrinsic graphene characterized by small Dirac points and high carrier mobility. The carrier mobility of the transferred graphene reaches 9090 cm^2^ V^−1^ s^−1^, demonstrating a superior carrier mobility over that from other dry transfer methods. In a nutshell, the proposed clean and efficient transfer method holds great potential for future applications of graphene.

## Graphical abstract

In the Cu_2_O‐Electric‐field Ice Transfer (CEIT) method, ice serves as a pollution‐free transfer medium, the electric field enhances adhesion force between ice and graphene, and Cu_2_O reduces the adhesion force between graphene and copper. The CEIT method successfully transfers graphene to various substrates, achieving a carrier mobility of 9090 cm^2^ V^−1^ s^−1^. This method shows great potential for future applications of graphene.

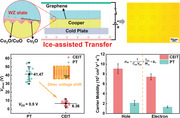

## 1 Introduction

The development of simple, universal, and reliable graphene transfer technologies is crucial for realizing the full potential of graphene in various applications, including but not limited to flexible thin‐film electronics, energy storage, and sensors (like electronic skin, organic photovoltaic cells, and gas sensors). Various strategies have so far been developed for the synthesis of graphene films. Among them, chemical vapor deposition (CVD) has commonly been employed for the synthesis of large‐area and high‐quality graphene films.^[^ ^1^, ^2^ ^]^ However, graphene films fabricated through the CVD method are typically grown on metallic substrates (such as Cu, Ni, and Pt),^[^ ^3^ ^]^ thereby requiring transfer to other substrates for further processing and applications. For example, graphene was transferred to a silica substrate to make electronic devices,^[^ ^4^ ^]^ or transferred to a polydimethylsiloxane (PDMS) substrate to make flexible sensors.^[^ ^5^, ^6^ ^]^

Two main types of transfer methods consisting of wet transfer and dry transfer have been widely used. Wet transfer involves the removal of the metallic substrate through chemical etching, resulting in isolated graphene films. In this method, a polymer support layer (usually poly methyl methacrylate (PMMA)) was commonly utilized to safeguard the detached graphene film to prevent its damage due to surface tension. However, the PMMA enabled transfer (PT) process often left polymer residues that weaken the electronic and optical properties of graphene. Additionally, it generated a significant amount of etching solutions detrimental to its scalability. To address these challenges, materials such as paraffin,^[^ ^7^ ^]^ rosin,^[^ ^4^ ^]^ and other small molecule substances^[^ ^8^ ^]^ have been used as alternatives to the PMMA support layer. However, the generation of large volumes of etching solutions is still problematic. Alternatively, dry transfer using solid elastomers as support layers is advantageous in terms of eliminating the need to etch the original substrate and avoiding the generation of etching solutions, making dry transfer a promising route for industrialization. However, graphene films exhibiting strong adhesion force to the original substrate during this process hinder the application of this technology,^[^ ^9^ ^]^ leading to incomplete transfer of CVD‐grown graphene.^[^ ^10^ ^]^ Additionally, the formation of polymer residues can be more severe in dry transfer than in wet transfer. Thus, various strategies have been proposed to improve dry transfer, including temperature control^[^ ^11^ ^]^ and support layer substitution.^[^ ^12^ ^]^ However, these approaches are still limited in terms of polymer contamination during heating and pressing processes.^[^ ^13^ ^]^ To address these challenges, clean support layers, such as ice^[^ ^14^ ^]^ or water^[^ ^15^, ^16^ ^]^ have been introduced in the dry transfer method to yield minimum pollution but limited to the transfer of 2D graphene from smooth substrates, such as mica and silicon. Therefore, these methods are ineffective for the transfer of graphene films grown on metal substrates by the CVD method due to the rough surface and strong adhesion force of metal substrates to the graphene films.

Herein, a novel ice‐enabled transfer method under an applied electric field and in the presence of Cu_2_O (Cu_2_O‐Electric‐field Ice Transfer, abbreviated CEIT) was proposed. The underlying principles of CEIT consisted of first applying an electric field to enhance the wettability of the rough surface with water molecules to achieve a complete transfer. The second principle consisted of using Cu_2_O to reduce adhesion force between graphene and the original substrate for separating graphene from metal substrates. Experimentally, the proposed CEIT method successfully transferred CVD‐synthesized graphene films onto various substrates, including PDMS, Teflon, C_4_F_8_, and SiO_2_. Notably, graphene films transferred through CEIT exhibited better smoothness, completeness, and absence of pollution when compared to the traditional transfer method. Key outcomes of the CEIT method included enhanced electrical properties of the transferred graphene. For example, field‐effect transistors fabricated by CEIT‐method graphene displayed near‐zero Dirac voltage and carrier mobility up to 9090 cm^2^ V^−1^ s^−1^, ≈3‐fold higher than that of PT‐method graphene. In addition, CEIT enabled the production of both patterned and large‐area graphene films, showcasing its versatility for various applications. In a nutshell, the as‐proposed CEIT method offered a clean and efficient transfer process of CVD‐grown graphene, with the potential for widespread large‐scale applications.

## 2 Results and Discussion

### 2.1 Basic Work Principle of CEIT

**Figure 1:**
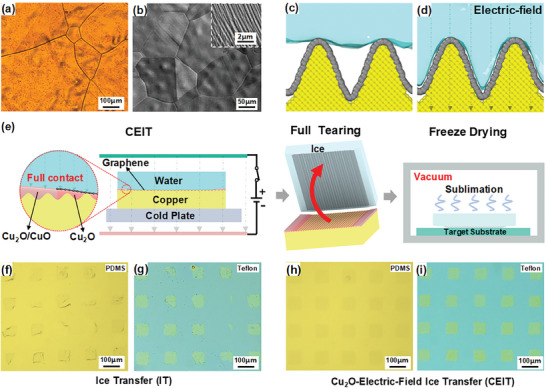
a) Optical micrograph of copper‐substrate graphene. b) SEM image of copper‐substrate graphene. c) Molecular dynamics of water wetting behavior on a rough copper‐substrate graphene surface, d) Wetting behavior under electric field. e) Flowchart of the CEIT method. f,g) Transferred patterned graphene via the IT method. h,i) Transferred patterned graphene via the CEIT method.

The success in the ice‐enabled dry transfer of graphene relied on the full contact of the ice/graphene interface to ensure the fabrication of undamaged graphene films. **Figure** **1**a,b highlights the challenges of graphene on a rough copper substrate monitored through optical microscopy and scanning electron microscopy (SEM). At the microscopic level, the critical question was whether water molecules were able to fully wet such uneven surfaces, constituting a crucial factor for establishing ample contact area and sufficient adhesion force at the ice/graphene interface. Molecular dynamics (MD) simulations were conducted to clarify this issue. Figure 1c summarizes the results of our MD simulation, in which water molecules are challenging in wetting the microstructured surfaces of copper‐substrate graphene. Built on our earlier studies,^[^ ^17^ ^]^ an external electric field was used to enhance the wetting of water molecules on various microstructured surfaces of the water layer. As illustrated in Figure 1d, the introduction of an electric field enabled water molecules to thoroughly permeate the microstructures of the graphene surface on the copper substrate, leading to increased contact area and adhesion force between ice and graphene. Another critical question was that the adhesion force of graphene to the original substrate did not surpass the intrinsic strength of graphene itself to ensure the fabrication of undamaged graphene films. To modulate the adhesion force between graphene and the original substrate, Cu_2_O was inserted at the interface between graphene and the substrate to reduce the adhesion force and effectively minimize the risk of graphene tearing during the transfer process. The culmination of these strategies resulted in an efficient CEIT method.

A schematic diagram of the ice‐enabled graphene transfer using the CEIT method is displayed in Figure 1e. Ice was exclusively employed as a support medium due to its eco‐friendly nature. During the CEIT procedure, copper‐based graphene was first oxidized to form Cu_2_O around and at the bottom of graphene. The introduction of Cu_2_O under graphene reduced the adhesion force between graphene and the copper substrate, while the introduction of an electric field ensured the establishment of a tightly bonded state between the water and graphene, boosting the adhesion force between the ice layer and graphene. The synergistic utilization of Cu_2_O and electric field facilitated a clean and damage‐free transfer of graphene. Additionally, a cold plate was utilized to convert the water layer into ice to initiate the freezing process at the water‐graphene interface. This approach prevented the detrimental expansion of ice at the ice‐graphene interface (Detailed explanation is shown in Figure S1, Supporting Information). Following the freezing process, a robust adhesion was established between the ice and graphene. By disrupting the copper‐ice structure, the entire graphene film was able to adhere to the ice medium without leaving any residue on the copper substrate, thereby accomplishing full transfer of graphene. In comparison, the Ice‐Enabled Transfer (IT) method (Figure S2, Supporting Information) without an electric field and Cu_2_O showed lower efficacy when compared to CEIT. During the removal of the ice medium through freeze‐drying, an ice layer was directly transformed from solid to gaseous state, bypassing the liquid water phase. This step was critical since high surface tension of liquid water may compromise the quality of the transferred graphene. Figure 1f‐i illustrates the results of transfers to the target substrates using IT (Figure 1f,g) and CEIT (Figure 1h,i) methods, respectively. Obviously, the CEIT method successfully transferred patterned graphene samples onto various substrates, including Teflon and PDMS with nearly 100% transfer efficiency. By comparison, the IT method without additional assistance achieved a considerably lower transfer efficiency with a lot of breakage and wrinkles.

The effectiveness of the CEIT method also relies on controlling the adhesion force at the ice/graphene and graphene/substrate interfaces. Specifically, the adhesion force of graphene to the original substrate should not surpass the intrinsic strength of the graphene itself, while the adhesion force between the ice and the graphene must surpass that between graphene and original substrate. To achieve these conditions, Cu_2_O and external electric field were combined in the CEIT strategy. Density functional theory (DFT) calculations were employed to unveil the role of Cu_2_O in adjusting the adhesion force across Cu/graphene, Cu_2_O/graphene, SiO_2_/graphene, and ice/graphene. As depicted in **Figur** **e** **2**a, the adhesion force of Cu/graphene generally outstripped that of the other interfaces. However, the adhesion force of ice/graphene surpassed those of Cu_2_O/graphene and SiO_2_/graphene, while being weaker than that of Cu/graphene. Consequently, the supporting ice layer adeptly separated graphene from the SiO_2_ and Cu_2_O but failed to achieve separation from the copper substrate.

**Figure 2:**
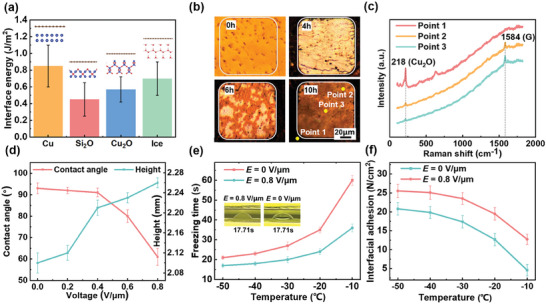
a) Surface energies of graphene on different substrates (DFT calculation results). b) Results of Cu_2_O interlayer growth for different durations. c) Raman characterization of graphene on oxidized copper substrate. d) Contact angle and height changes of droplets under different electric fields. e) Influence of electric field and temperature on freezing time of droplet. The illustration shows experimental images with or without an electric field. f) Influence of electric field and temperature on adhesion force of ice/graphene.

Fortunately, the adhesion force between graphene and copper can be mitigated by introducing Cu_2_O into the graphene/copper interface through the hydrothermal oxidation method.^[^ ^18^, ^19^ ^]^ A copper‐substrate patterned graphene sample (50 × 50 µm^2^) exposed to varying duration of the hydrothermal oxidation process is presented in Figure 2b, and a detailed oxidation process of graphene/copper is provided in Figures S3 and S4 (Supporting Information). Obviously, the oxidation process formed Cu_2_O with distinct colorations for Cu (yellow) and Cu_2_O (red). As the oxidation process progressed, Cu_2_O accumulated within the Cu/graphene interface, altering the color of the copper from yellow to red. In areas not covered by graphene, the copper surface was further oxidized, forming a mixture of Cu_2_O and CuO, resulting in a darker or even black color on the periphery of graphene. The Raman spectra at various points of a 10 h oxidation process are displayed in Figure 2c. Point 1, located outside the patterned graphene region (as shown in Figure 2b), exhibited strong Raman signal at 218 cm^−1^, indicating high concentration of Cu_2_O.^[^ ^18^, ^19^ ^]^ Since this point was not covered by graphene, no G‐peak (≈1580 cm^−1^) was observed. After prolonged oxidation, both the edge (Point 2) and the center (Point 3) of the patterned graphene region showed faint signals of Cu_2_O, indicating progressive infiltration of Cu_2_O into the graphene/copper interface. Simultaneously, the Raman spectra at points 2 and 3 exhibited pronounced G‐peaks but lacked obvious D‐peaks, confirming the intactness of the graphene's carbon ring structure. Thus, the hydrothermal oxidation method integrated Cu_2_O at the graphene/copper interface without damaging the graphene films.

The adhesion force at the ice/graphene interface was then optimized through electrically assisted method. The changes in contact angle and shape of the water droplet on a copper‐based graphene surface under different electric fields are shown in Figure 2d (experiment shown in Figure S5, Supporting Information). Lower water contact angles indicate improved wettability, which is indicative of a potential increase in adhesion force at the ice/graphene interface.^[^ ^20^, ^21^ ^]^ A smaller contact angle signified superior wettability, allowing water molecules to wet a larger extent of surface microstructures. Such enhancement in wettability increased the contact area between the ice and substrate after freezing, reinforcing the adhesion force. Copper‐based graphene is typically hydrophobic, with a surface contact angle reaching 90°. By comparison, monolayer graphene possesses wetting transparency,^[^ ^22^ ^]^ causing minimal alterations to the wettability of a substrate. Similar to the pure copper substrate, graphene on copper substrate was hydrophobic, hindering complete wetting of the microstructures by water. The introduction of electric field would drastically alter these properties. Under an external electric field of 0.8 V µm^−1^, water droplets were stretched, leading to an increased droplet height from 2.12 to 2.32 mm by 10%. Such elongation generated pushing force at the water‐substrate interface, encouraging more complete wetting of the water molecules on the copper‐based graphene surface. Simultaneously, the contact angle decreased from 93° to below 61°, further enhancing the wettability.^[^ ^17^ ^]^ Furthermore, numerous studies suggested that the application of external electric fields can facilitate ice growth.^[^ ^23^, ^24^, ^25^, ^26^, ^27^ ^]^ As depicted in Figure 2e, the presence of an external electric field accelerated the freezing process by >20% compared to conditions in the absence of electric field (Figure S6, Supporting Information).

The effects of these factors in adjusting the adhesion force at the ice/graphene interface are given in Figure 2f. The interfacial force test method is described in detail in Section 1 and Figure S7 (Supporting Information). The adhesion strength at the ice/graphene interface was enhanced in the presence of external electric field. In addition, decrease in the cooling plate temperature significantly increased the adhesion force at the ice/graphene interface^[^ ^28^, ^29^ ^]^ until stabilizing at −50 ^○^C. Temperature variations significantly influenced the interfacial adhesion force of ice due to the quasi‐liquid water phase near the substrate/ice interface.^[^ ^30^ ^]^ Intriguingly, such quasi‐liquid phase formed even at sub‐zero temperatures, which cannot be avoided.^[^ ^31^ ^]^ After a series of rigorous experiments, the macroscopic interfacial adhesion force under specific conditions (−50 ^○^C and external electric field intensity of 0.8 V µm^−1^) reached a maximum value of 26 N cm^−2^. These established parameters were used in the subsequent experiments.

### 2.2 Comparative Analysis on Transferred Graphene

**Figure 3:**
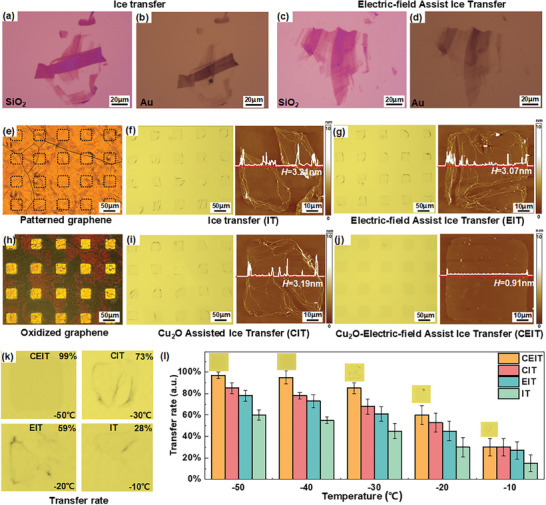
a,c) Mechanically exfoliated graphene on SiO_2_ substrate. b,d) Mechanically exfoliated graphene transferred to Au substrate. e) Unoxidized and h) oxidized copper‐substrate patterned graphene. Optical micrograph and AFM images of graphene transferred onto a PDMS substrate by the f) IT method, g) EIT method, i) CIT method, CEIT method. j) *H* is the average height of the line. k) Results of the different transfer rates using different transfer methods. l) The variation rules of transfer rates at different temperatures of different transfer methods.

The transfer outcomes of the CEIT method and its derived methods were also explored. **Figure** **3**a–d showcases the transferred results of mechanically exfoliated graphene from SiO_2_ to Au substrate through IT and Electric‐field Assisted Ice Transfer (EIT) methods. For smooth substrates like SiO_2_, the introduction of external electric field has a slight impact the adhesion force at the ice/graphene interface for flat surfaces without wetting issues. Thus, successful full transfer of graphene to the target substrate can be achieved irrespective of the external electric field, albeit with slight drifting, wrinkling, and missing issues. The challenge would arise when transferring graphene grown on copper substrate. The patterned graphene on a non‐oxidized rough copper substrate is depicted in Figure 3e, in which the grid denotes the invisible patterned graphene and both IT and EIT methods were employed for the transfer. In Figure 3f, graphene films transferred via the IT method exhibited incompleteness and significant damage, as evidenced by the voids observed by Atomic Force Microscopy (AFM). Therefore, the IT method failed to achieve a complete graphene film transfer. By comparison, the EIT method under an assisted electric field improved the transfer but still showed defects in both optical microscopy and AFM images (Figure 3g). These defects hindered the accurate determination of single‐layer graphene thickness. For instance, the measured thickness (*H* = 3.07 nm) significantly exceeded the expected values for a single layer of graphene.

Patterned graphene on oxidized rough copper substrate is presented in Figure 3h. Color changes were observed due to the oxidation process, resulting in enhanced visibility of the patterned graphene films. With the assistance of Cu_2_O, the graphene transferred by Cu_2_O Assisted Ice Transfer (CIT) method (Figure 3i) was comparable to that by the EIT method. Despite the transfer of most patterned graphene films to the target substrate, significant defects persisted in the graphene films, hindering AFM from accurately determining the thickness of the single‐layer graphene. By comparison, the CEIT method, combining both Cu_2_O and an electric field, substantially outperformed all other mentioned methods. In Figure 3j, the graphene films appeared virtually undamaged, achieving a complete transfer of patterned graphene. The absence of defects on the graphene film allowed relatively accurate measurement of the thickness of the single‐layer graphene by AFM. From Figure 3j, a thickness of *H* = 0.91 nm was measured, very close to that of single layer graphene, aligning with previous reports.^[^ ^32^ ^]^ Additional AFM images of the CEIT method are available in Figure S8 (Supporting Information), which further demonstrate its superiority. Figure S9 (Supporting Information) shows the carryover of oxide of copper particles that may occur during the transfer process; this issue can be resolved with an HCl solution.

For quantitative comparison, we use the transfer rate to evaluate the efficiency of the transferred graphene. Transfer rate is defined as the area of the transferred graphene sheet divided by the area of the complete graphene sheet and Figure 3k shows graphene films with different transfer rate. The average transfer rate for 20 graphene sheets within a region was estimated, and Figure 3l shows the average transfer rate under different conditions. Obviously, the CEIT method approached a nearly 100% transfer rate at −50 ^○^C, implying that the CEIT technique can transfer almost all patterned graphene under optimal conditions. Besides patterned graphene, the CEIT method has successfully facilitated the transfer of larger graphene film, as demonstrated by the large‐area transfer results and characterizations shown in Figures S10 and S11 (Supporting Information). The CEIT method enables the transfer of large‐area graphene films that are clean, high‐quality, and intact, with a maximum size of 400 × 600 µm in a rectangular area. In contrast, graphene transferred by the PT method is not only heavily contaminated but also extensively damaged. Therefore, the CEIT method notably outperformed the traditional PT method in terms of transfer quality and cleanliness.

**Figure 4:**
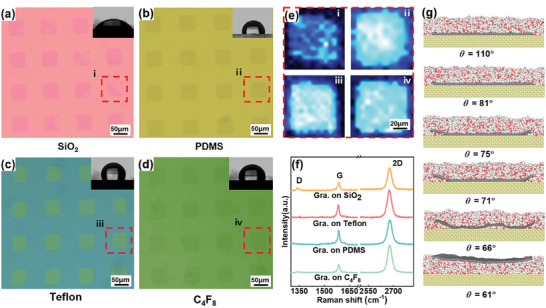
Patterned graphene transferred to a) SiO_2_ substrate, b) PDMS substrate, c) Teflon substrate, and d) C_4_F_8_ substrate. e) G‐peak intensity of Raman mapping for patterned graphene. f) Raman single‐point characterization of graphene with different target substrates. g) Molecular dynamics computational modeling exploring the effect of wettability on the adhesion process of graphene on target substrate. *θ* is the contact angle of the target substrate.

As abovementioned, mechanically transferred exfoliated graphene from SiO_2_ to gold substrate through the IT method was limited by slight drifting, wrinkling and missing issues, despite achieving an almost complete transfer. Interestingly, when attempting to transfer CVD graphene by the CEIT method onto a PDMS substrate, there were no such issues. However, changing the target substrate to SiO_2_ resulted in such issues of graphene transfer (**Figure** **4**a). Previous studies indicated that wrinkling and drifting would be inevitable for water encountering 2D materials, and thinner graphene exacerbated these issues.^[^ ^33^ ^]^ Using the CEIT method even at sub‐zero temperatures, a quasi‐liquid layer persisted at the ice/graphene interface. Additionally, a film of condensed water formed during the CEIT process on the target substrate, especially for hydrophilic substrates. The surface tension of the water can induce drifting, wrinkling, and missing of graphene after the formation of a water layer at the graphene interface. Therefore, a highly hydrophilic target substrate may condense a significant number of water molecules at the graphene/target‐substrate interface, greatly impeding the high‐quality transfer of graphene.

The transferred graphene on four target substrates SiO_2_, PDMS, Teflon, and C_4_F_8_ is compared in Figure 4a–d. SiO_2_ exhibited the strongest hydrophilicity with a contact angle of 60° (Figure 4a). Consequently, a film of condensed water formed on the SiO_2_ substrate, potentially causing drifting, wrinkling, or even resulting in missing areas of the transferred graphene film. By contrast, the other three hydrophobic substrates (PDMS, Teflon, and C_4_F_8_) with contact angles beyond 120° did not exhibit such issues (Figure 4b–d). The graphene transferred onto hydrophobic substrates showcased structural arrangement, smoothness, and integrity. The differences across various substrates were further highlighted using representative graphene films characterized by Raman spectroscopy. Figure 4e presents the Raman spectrum of the square graphene film, showing G‐peak intensity. In Figure 4e(ii)–(iv) (substrates PDMS, Teflon, and C_4_F_8_), distinct uniformity was exhibited, in stark contrast with the damaged and non‐uniform Raman spectra of the graphene flake in Figure 4e(i) (substrate: SiO_2_). The quality of graphene transferred onto hydrophobic substrates appeared markedly higher than that transferred onto hydrophilic substrates, emphasizing the significant impact of the target substrate's wettability on the transferred graphene. Figure S12 (Supporting Information) shows that the Teflon‐coated SiO_2_ is treated with oxygen plasma and it has a contact angle range of 50°–130°, which visually shows the effect of different wettability on the transfer results. More hydrophilic surfaces result in poorer transfers, while more hydrophobic surfaces yield excellent transfer results. The single‐point Raman characterization in Figure 4f also supported this observation. Upon transfer onto the three hydrophobic substrate surfaces, the D‐peak of graphene became less conspicuous or even completely absent, which means the transferred graphene is of high quality, in sharp contrast to the low‐quality graphene for transfer onto hydrophilic SiO_2_ substrate surfaces.

To gain a better understanding, MD simulations were performed by placing a graphene film on the target substrate enveloped by a thin layer of water molecules. The water molecules were used to simulate the condensed water layer produced during the CEIT process. Surfaces with varying contact angles were then achieved by adjusting the parameters (Figure 4g). Obviously, as the contact angle decreases, driven by the hydrophilic interactions between water and the substrate, the spreading of water becomes stronger. As expected, turning the water‐substrate interaction to highly hydrophilic (contact angle *θ* ≤ 61°, Figure 4g) resulted in the spontaneous infiltration of water molecules into the interface between graphene and target substrate, leading to the drifting, wrinkling, or even missing of the graphene film. Adjustment of the interaction between water and the substrate to more hydrophobic (contact angle *θ* ≥ 110°) inhibited infiltration of water molecules into the interface between graphene and the substrate with the graphene remaining on the substrate. Therefore, MD simulations elucidated the impact of the target substrate's wettability on the results of graphene transfer. Consequently, the CEIT method achieved superior performance with hydrophobic target substrates. For hydrophilic substrates, multiple transfer attempts might be necessary to attain optimal transfer outcomes. This issue could also be addressed by developing novel methods to avoid the presence of interfacial water.

### 2.3 Electrical Properties of Transferred Graphene by CEIT

**Figure 5:**
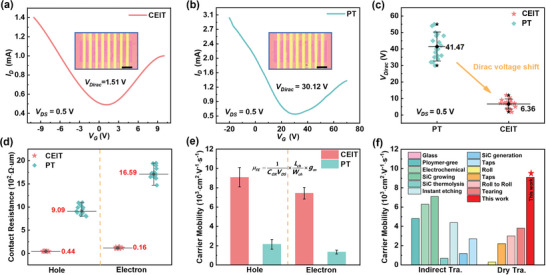
Transfer characteristic curves of graphene transferred by a) CEIT method and b) PT method. The illustration shows local images of FETs. The scale bar is 25 µm. The channel length *L_ch_* is 8 µm and channel width *W_ch_* is 50 µm. c) Comparison of Dirac voltages of graphene transferred by CEIT and PT methods. d) Comparison of contact resistances of graphene transferred by CEIT and PT methods. e) Comparison of carrier concentrations of graphene transferred by CEIT and PT methods. f) Comparison of the present work with previous works.^[^ ^34^, ^35^, ^36^, ^37^, ^38^, ^39^, ^40^, ^41^, ^42^ ^]^ The aforementioned electrical properties were measured before the HCl solution rinse.

A series of graphene field effect transistors (FETs) were prepared for assessing the electrical performances of transferred graphene by CEIT method. The schematic and actual circuit diagrams of the FETs are presented in Figure S13 (Supporting Information). The electrical properties of single‐layer graphene transferred by CEIT and PT methods are displayed in **Figure** **5**a,b, respectively. Obviously, graphene transferred by the CEIT method demonstrated significantly lower Dirac voltage (*V_Dirac_*), indicating higher quality. Here, Dirac voltage is defined as the specific reverse bias voltage for the device's conductivity reaching minimum corresponding to the Dirac point or charge neutrality point where the conduction and valence bands of the graphene film intersect. For zero gate voltage, the intrinsic graphene exhibited the lowest conductivity. Consequently, *V_Dirac_* served as a potent indicator, efficaciously reflecting the inherent traits of graphene films. The *V_Dirac_* distributions of several graphene transistors prepared by CEIT and IT methods are presented in Figure 5c. The *V*_Dirac_ voltage of graphene transferred by PT method ranged from 30.12 to 56.44 V, with an average value of 41.47 V. Conversely, the *V*_Dirac_ distribution of graphene transferred by CEIT varied between 1.51 and 11.25 V, with an average value of 6.36 V. The lower mean value of *V_Dirac_* suggested a closer alignment of the electrical traits of graphene transferred by CEIT with those of intrinsic graphene when compared to the graphene transferred via PT method. The raw data corresponding to the output and transconductance curves are provided in Figure S14 (Supporting Information).

In Figure 5d, the contact resistance of FETs fabricated via the CEIT method was lower regardless of the type of charge carrier, holes, or electrons. Less contact resistance suggested a cleaner surface of graphene transferred by the CEIT method, implying higher quality. Figure 5e compares the field effect mobility (*µ_FE_*) of FET prepared by both transfer methods. *µ_FE_* can be determined as follows:

(1) $$\mu_{FE}=\frac{1}{C_{ox}V_{DS}}\times \frac{L_{ch}}{W_{ch}}\times \frac{\Delta I_{DS}}{\Delta V_{G}}$$

where *L_ch_* represents channel length, *W_ch_* is channel width, *C_ox_* refers to the gate capacitance, *I_DS_* is the drain‐to‐source current, *V_DS_* denotes the drain voltage, and *V_G_* is the back‐gate voltage.

For graphene transistors transferred by PT method, the electron mobility was determined as 2558 cm^2^ V^−1^ s^−1^, with an error bar of 501 cm^2^ V^−1^ s^−1^, while the hole mobility was determined as 1257 cm^2^ V^−1^ s^−1^, with an error bar of 205 cm^2^ V^−1^ s^−1^. For transistors prepared using the CEIT method, the electron mobility was estimated to be 9090 cm^2^ V^−1^ s^−1^, with an error bar of 907 cm^2^ V^−1^ s^−1^, while the hole mobility was 7454 cm^2^ V^−1^ s^−1^, with an error bar of 603 cm^2^ V^−1^ s^−1^. It is noted that the electrical properties of transferred graphene sheets were measured before HCI solution rinse. The electrical properties of transferred graphene after HCl solution rinse are presented in Figure S15 (Supporting Information). The measured electron mobility is 9033 ± 883 cm^2^ V⁻¹ s⁻¹, indicating that HCl solution rinse has rarely impact on the electrical properties of the transferred graphene. Therefore, for FETs prepared by the CEIT method, both the calculated electron and hole mobilities were higher than those prepared by the PT method. Figure 5f shows a comparison of electrical performance between the CEIT‐method graphene and recently reported graphene obtained by indirect and dry transfer methods.^[^ ^34^, ^35^, ^36^, ^37^, ^38^, ^39^, ^40^, ^41^, ^42^ ^]^ It can be found that the graphene prepared by CEIT exhibits highest carrier mobility compared to the reported results ranging from 308–7100 cm^2^ V^−1^ s^−1^, demonstrating the capability of transferring high‐quality graphene using the CEIT process.

## 3 Conclusion

A novel, defect‐free, and highly efficient graphene CEIT method was developed. By leveraging external electric field and Cu_2_O interlayers, the CEIT method facilitated the transfer of CVD‐grown graphene films. The electric field played a crucial role in ensuring thorough wetting of the graphene's surface, while the introduction of Cu_2_O reduced the adhesion force between the graphene and its original substrate. Notably, ice served as the medium during the transfer process, eliminating the need for polymers or organic solvents. The graphene transferred by the CEIT method exhibited elevated cleanliness, planarity, and enhanced electrical characteristics than traditional methods, showcasing an impressive electron mobility value reaching 9090 cm^2^ V^−1^ s^−1^. The versatility of the CEIT method extended to a wide range of target substrates, from rigid semiconductors to flexible polymers, with particularly observed superior transfer effects on hydrophobic substrates. Overall, the proposed innovative ice‐based transfer and cleaning technique has the potential in developing ideal graphene surfaces, interfaces, heterostructures, and large‐scale array sensors. It may also revolutionize the large‐scale manufacturing and integrated application of monolayer graphene.

## 4 Experimental Section

### CEIT Method

First, copper‐based graphene underwent photolithographic patterning to create patterned graphene sheets, as detailed in Section 2 of the Supporting Information. This patterned graphene was then subjected to hydrothermal method in hot water at 95 °C for 10 h to develop Cu_2_O at the graphene/copper interface, making them suitable for CEIT. The freezing process, illustrated in Figure S16 (Supporting Information), began with positioning a cold plate and electrode plates in a 0 °C chamber adjacent to the freeze dryer. The copper‐based graphene was fixed on the electrode plate. The water was dropped on the cold plate. Simultaneously, an external electric field of 0.8 V µm^−1^ was applied between two electrode plates. A high‐voltage power supply (Trek 610E) was used as the voltage source, equipped with robust overvoltage protection capabilities to ensure the safe conduct of experiments. The −50 °C electrode‐plate acted as a thermal buffering, keeping the ice at a low temperature, as the copper‐based graphene was transferred to the adjacent freeze dryer. To avoid ice melting during the transfer process, the copper‐based graphene with the electrode plates was rapidly transferred into the freeze dryer in a couple of seconds. Concurrently, the target substrate was precooled in the freeze‐dryer to −50 °C to avoid any melting from temperature discrepancies upon contact. The subsequent steps were executed in the −50 °C environment of the freeze‐dryer. The detaching process is detailed in Figure S17 (Supporting Information). After detachment, the ice column/graphene assembly was placed on the target substrate for freeze‐drying. The parameters of freeze‐drying are listed in Table S2 (Supporting Information). The freeze dryer utilized was the LGJ‐10FD model from Beijing Songyuan. Upon completing the freeze‐drying, the graphene was successfully transferred to the target substrate. Any oxide of copper residue on the target substrate could be removed by rinsing with a 2.5% HCl solution.

### Molecular Dynamics

MD simulations were conducted using the Large‐Scale Atomic/Molecular Massively Parallel Simulator (LAMMPS).^[^ ^43^ ^]^ To this end, a monolayer graphene sheet with dimensions of 5 × 5 nm^2^ was positioned over a solid substrate immersed in a 1.5 nm water film consisting of 4009 water molecules. Substrates possessed Diamond Cubic Structure, and water molecules were modeled using the Single Point Charge/Extended (SPC/E) model.^[^ ^44^ ^]^ The atomic interactions between carbon atoms in the graphene sheet were described by the AIREBO potential. The 12–6 Lennard‐Jones (LJ) potential was used to describe the atomic interactions between water and the substrate, as well as between graphene and the substrate. The non‐bonded interaction functions can be expressed as follows:

(2) $$U_{ij}=\frac{q_{i}q_{j}}{r_{ij}}+4\varepsilon_{ij}\left[{\left(\frac{\sigma_{ij}}{r_{ij}}\right)}^{12}-{\left(\frac{\sigma_{ij}}{r_{ij}}\right)}^{6}\right]$$

where *U_ij_* represents the non‐bonded potential energy of the *i‐*th and *j‐*th atoms. *q_i_* and *q_j_* are the charges of the *i‐*th and *j‐*th atoms, respectively. *r_ij_* refers to the distance between the *i‐*th and *j‐*th atoms. *ε* and *σ* are the LJ well depth and LJ radius, respectively.

The LJ parameters of non‐bonded interactions are listed in Table S1 (Supporting Information).^[^ ^45^, ^46^, ^47^ ^]^ The simulation box size consisted of 217.2 × 108.6 × 64 Å^3^. PBCs were applied in the planar *x* and *y* directions of graphene, while a wall‐boundary condition was used in the off‐plane *z* direction. All MD simulations were performed under the NVT ensemble with a timestep of 1 fs. The temperature of the as‐investigated system was controlled using the Nose−Hoover thermostat technique.

### Density Functional Theory Calculations

DFT calculations were performed through Atomic‐orbital Based Ab‐initio Computation at UStc (ABACUS) package.^[^ ^48^, ^49^, ^50^ ^]^ The generalized gradient approximation (GGA) in the form of the Perdew–Burke–Ernzerhof (PBE) was employed for the exchange‐correlation function. The kinetic energy cutoff of the plane wave basis set was set to 100 Ry. Periodic boundary conditions (PBCs) with a 4 × 4 × 4 k‐point mesh was utilized. The Gaussian smearing method with a width of 0.01 eV was applied. The electronic iteration convergence threshold was set to be 10^−6^ eV, and structural relaxations were carried out with the forces exerted on each atom <0.01 eV Å^−1^. The stress threshold during cell relaxation was set at 0.05 GPa.

### PMMA Enabled Transfer

Initially, a layer of PMMA was covered onto the graphene synthesized on copper foil through spin processing at 2500 rpm. The graphene covered by PMMA was subsequently baked at 80 ^○^C for 1 h. Afterward, the sample was floated atop a copper etchant (NaOH solution) for 300 min to remove the growth substrate. The obtained PMMA‐supported graphene film was then thoroughly rinsed with deionized water, and the target substrate was brought into contact with the PMMA‐supported graphene film and dried by a nitrogen gun. The sample was then immersed in acetone at room temperature for 6 h to dissolve the PMMA support layer, followed by water rinse and drying by nitrogen stream. All process parameters of PT‐method in this study were drawn from previous reports.^[^ ^51^, ^52^ ^]^

### Graphene Characterization

The Raman spectra and maps were collected by a Renishaw system at the laser excitation wavelength and grating density of 532 nm laser line and 600 cm^−1^, respectively. To avoid laser‐induced heating, the laser beam size was set to be 500 nm and the laser power on the sample was adjusted to 0.75 mW. Atomic force microscopy (AFM) topography images were obtained in tapping mode.

### Fabrication of Back‐Gated Graphene Field Effect Transistors

The graphene synthesized on copper foil was initially transferred onto an oxidized silicon substrate featuring a 285 nm thick SiO_2_ layer using either the CEIT or PT method. The source/drain contacts on the graphene were carefully crafted by electron beam lithography, then metalized with a 20/30 nm layer of Ti/Au followed by a 12 h lift‐off process in room temperature acetone. Subsequently, the graphene film was crafted into a 50 µm wide ribbon using e‐beam lithography accompanied by oxygen plasma etching. All graphene field effect transistors maintained consistent dimensions throughout testing. The graphene channel and contact width were set to 50 µm, while the channel length varied between 5 and 50 µm. Titanium was employed as the electrode material due to its proven reliability in forming metal contacts with graphene.

## Conflict of Interest

The authors declare no conflict of interest.

## Author Contributions

H.M. and Y.H. designed the experiments. H.M., Y.H., K.W., S.C., Q.Z., and Y.W. performed the experiments and drew the diagram. X.C. provided resources. H.M., X.C., and J.W. wrote the manuscript. J.S. supervised the study. X.C., J.W., and J.Z. provided funding.

## Supporting information

Supporting Information

## Data Availability

The data that support the findings of this study are available from the corresponding author upon reasonable request.
