# Supplementary material for: Ice‐Enabled Transfer of Graphene on Copper Substrates Enhanced by Electric Field and Cu2O
Source: Adv Sci (Weinh). 2024 Jun 25;11(32):2402319. doi: 10.1002/advs.202402319 (PMC11348137; doi:10.1002/advs.202402319)
Supplement: Supplementary file 1 — Supporting Information [file ADVS-11-2402319-s001.docx]

Supporting Information

**Ice-Enabled Transfer of Graphene on Copper Substrates Enhanced by Electric Field and Cu_2_O**

Hechuan Ma^1^, Xiaoming Chen^1,2,^*, Yufei Han^1^, Jie Zhang^3^, Kaiqiang Wen^1^, Siyi Cheng^1^, Quanyi Zhao^1^, Yijie Wang^1^, Jianyang Wu^4,*^,Jinyou Shao^1^

^1^Micro- and Nanotechnology Research Center, State Key Laboratory for Manufacturing Systems Engineering, Xi’an Jiaotong University, Xi’an, Shannxi 710049, China

^2^XJTU-POLIMI Joint School of Design and Innovation, Xi’an Jiaotong University, Xi’an, Shaanxi, 710049, China

^3^Electronic Materials Research Lab, Key Laboratory of the Ministry of Education, Xi’an Jiaotong University, Xi’an, Shaanxi, 710049, China

^4^Department of Physics, Jiujiang Research Institute and Research Institute for Biomimetics and Soft Matter, Fujian Provincial Key Laboratory for Soft Functional Materials Research, Xiamen University, Xiamen 361005, China

*To whom correspondence should be addressed. Xiaomingchen@xjtu.edu.cn (X. Chen) and jianyang@xmu.edu.cn (J. Wu).

**1. Measurements of interfacial adhesion**

**Figure S7** displays images of the interfacial force testing setup. **Figure S7a** shows the ice-forming process. During this process, the square glass tube on the copper-based graphene was connected with a home-made holder. An external electric field of 0.8 V/µm was applied. **Figure S7b** shows the measuring process of the force between ice and graphene. The ice connected with the holder was lifted up by a hook of force-measuring equipment. Through recording the pulling force of hook, the interfacial strength between ice and graphene was estimated.

**2. Graphene patterning**

All graphene samples utilized in this study were sourced from Shenzhen Six Carbon Technology Company. The process of the patterning method is shown in **Figure S18a**. The graphene patterning process involves photolithography and oxygen plasma etching. Initially, a photoresist resin (EPG535) was spin-coated onto the copper-based graphene, using a speed of 6000 rpm. Due to the significant difference in Poisson's ratios between graphene and the photoresist, drying methods such as baking or thermal annealing were avoided to prevent damage to the graphene. Instead, the photoresist was dried in air for 24 hours. Photolithographic development was then employed to pattern the photoresist on the graphene surface. The exposed graphene was subsequently removed by oxygen plasma etching (60 W for 40 s), which oxidized the graphene to CO_2_, thereby leaving the patterned graphene under the photoresist. Finally, the photoresist was removed by soaking in acetone for 24 hours, resulting in clean, patterned graphene. In **Figure S18b** the SEM image shows the patterned graphene on a copper substrate, on which little residual contamination was observed.

**Figure S1.** (a) On the cold plate, the water was gradually frozen from the bottom to upwards. The space of unfrozen water along the glass tube served as an expansion buffer to prevent damage to the interface between ice and graphene. (b) The glass tube with water was placed in cold chamber of the freeze dryer. The freezing process was isotropic along various directions, where the expansion of ice occurred towards various directions without any buffer zones. The expansion from various directions can lead to the cracking of the ice, thereby damaging the interface between ice and graphene.


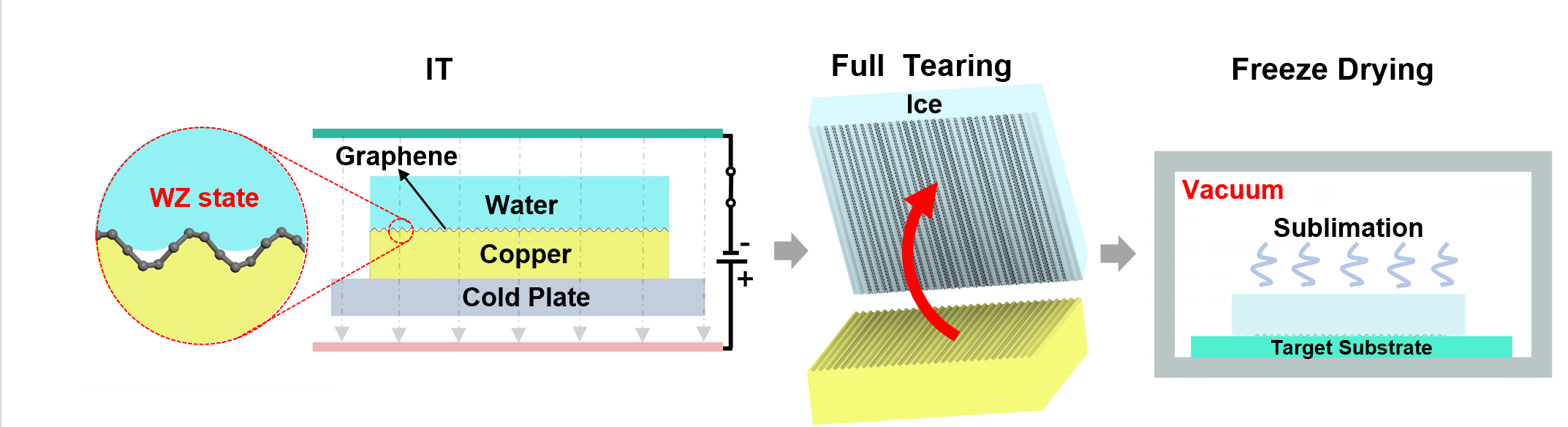


**Figure S2.** Schematic diagram of the IT method.


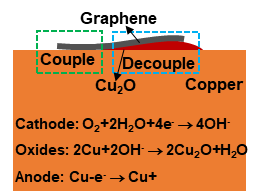


**Figure S3.** Schematic diagram of the generation of Cu_2_O.


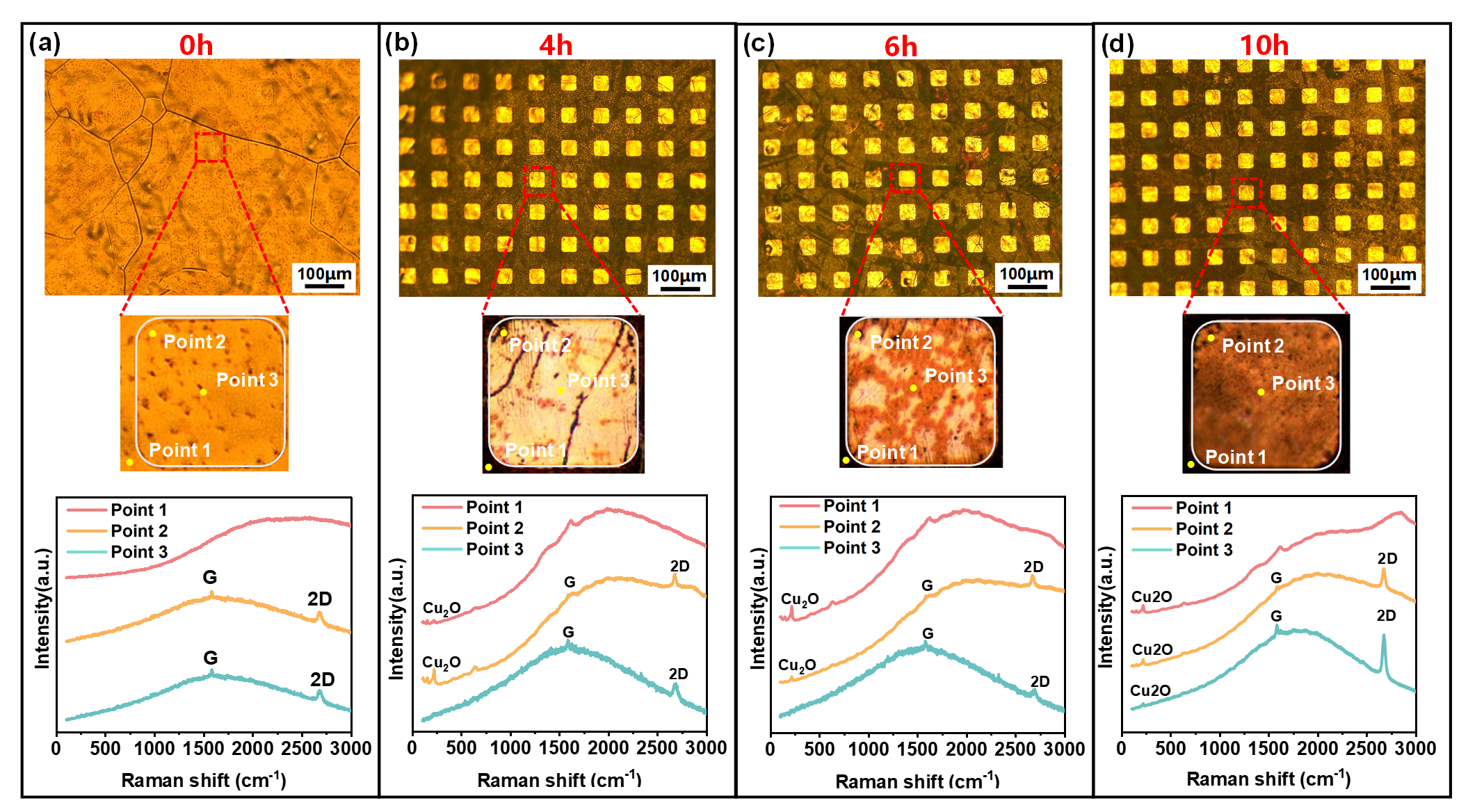


**Figure S4.** Photographs of Cu_2_O distribution and Raman spectroscopic at different locations under different hydrothermal durations: (a), (b), (c), and (d), corresponding to hydrothermal durations of 0, 4, 6, and 10 hours, respectively.


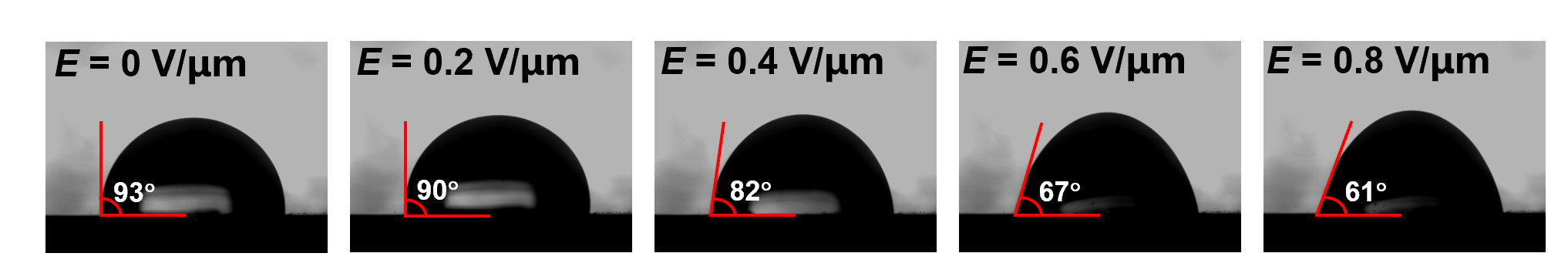


**Figure S5.** Angle change and droplet morphology under different electric fields.


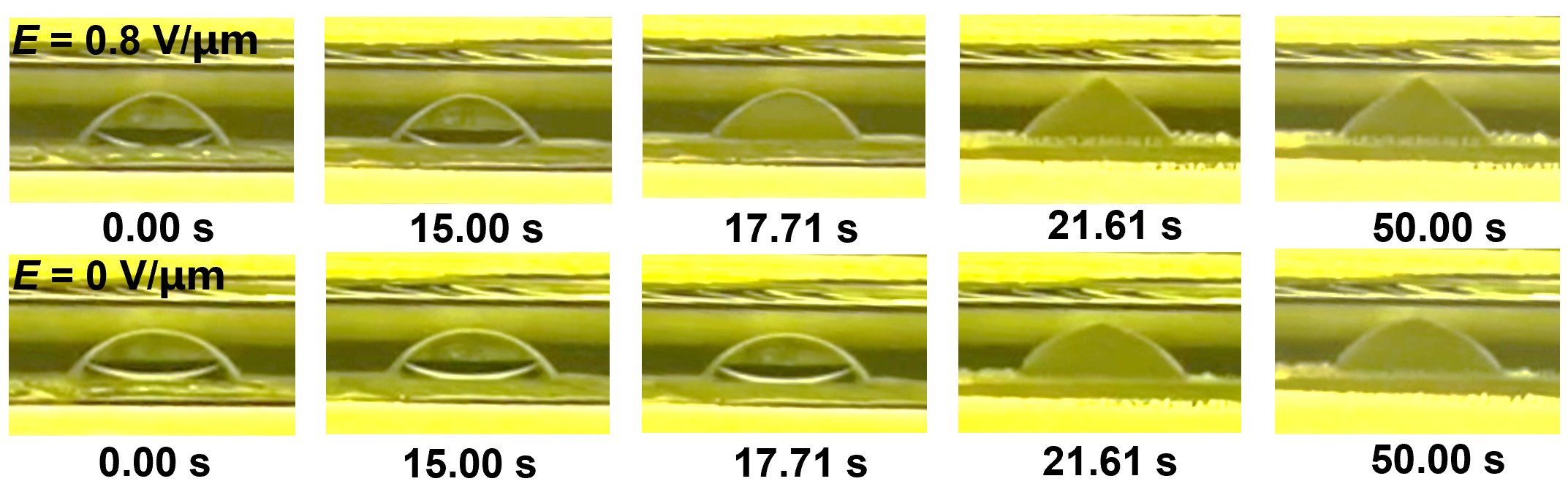


**Figure S6.** Ice formation time affected by external electric field.

**Figure S7.** (a) During the ice-forming process, the square glass tube on the copper-based graphene was connected with a home-made holder. An external electric field of 0.8 V/µm was applied. (b) The ice connected with the holder was lifted up by a hook of force-measuring equipment. Through recording the pulling force of the hook, the interfacial strength between ice and graphene was estimated.


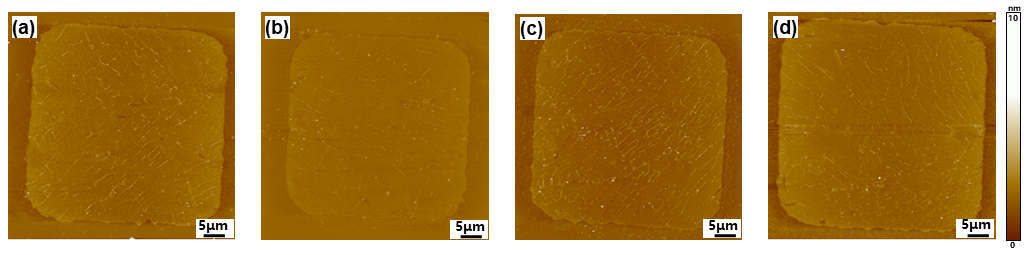


**Figure S8.** (a-d) AFM images of graphene transferred using the CEIT method cleaning by HCl solution, showing almost no white spots and very a clean surface.


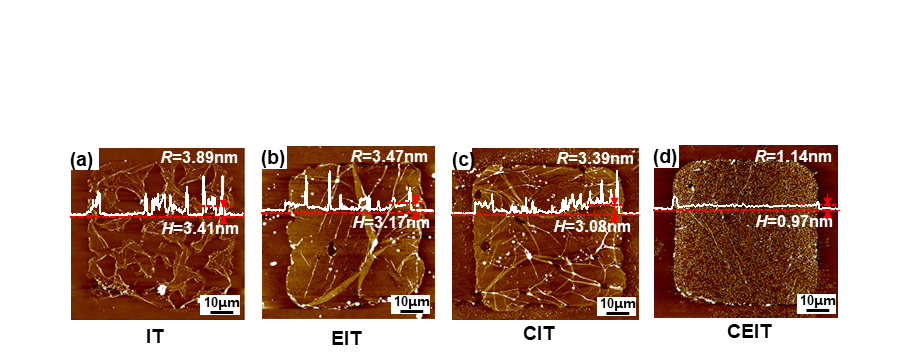


**Figure S9.** SEM image of CEIT transferred graphene without cleaning by HCl solution. (a-d) are IT, EIT, CIT, CEIT transferred graphene, respectively. There will be some white spots left on the surface. These white spots are remnants of copper oxide and can be removed using HCl solution.


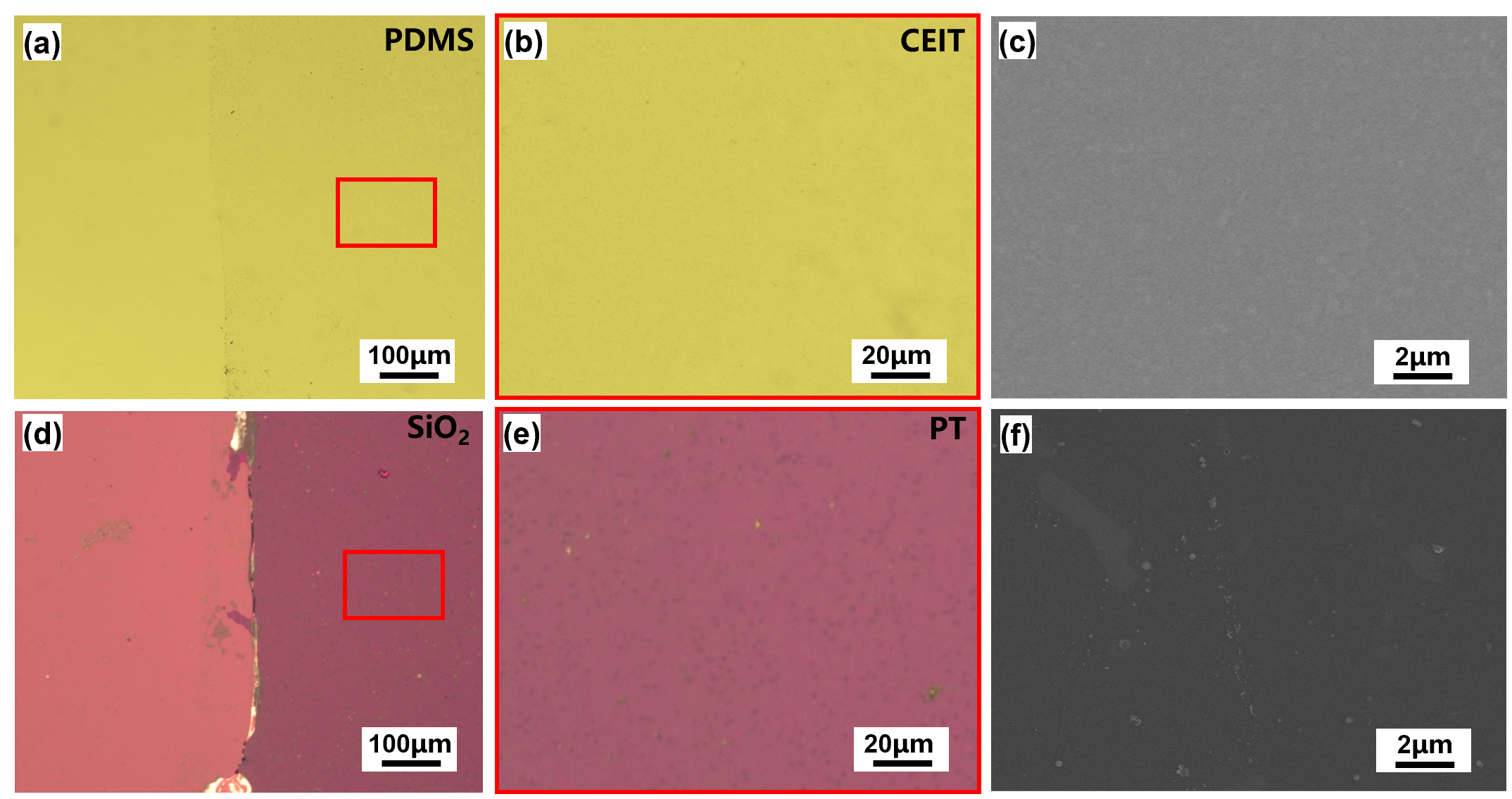


**Figure S10.** (a) Photomicrograph of graphene transferred to PDMS substrate by CEIT method. (b) Localized magnified photomicrograph. (c) Localized magnified SEM image. (d) Photomicrograph of graphene transferred to SiO_2_ substrate by PT method. (e) Localized magnified photomicrograph. (f) Localized magnified SEM image.

**Figure S11.** Raman characterization of graphene transferred by CEIT and PT methods.


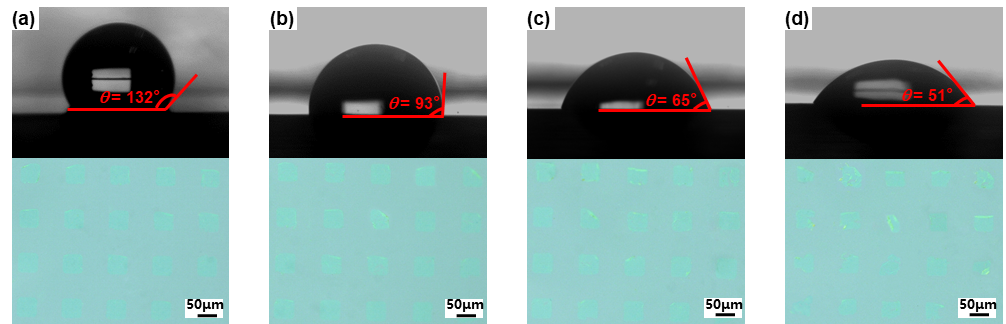


**Figure S12.** Transfer results at different contact angles: (a-d) Contact angles of surfaces treated with different oxygen plasma processes and transfer results under different contact angles. Surfaces with higher hydrophilicity lead to poorer transfers, while those with greater hydrophobicity yielded excellent results.

**Figure S13.** (a) Schematic diagram of electrical performance testing. (b) Actual circuit diagram of electrical performance testing.


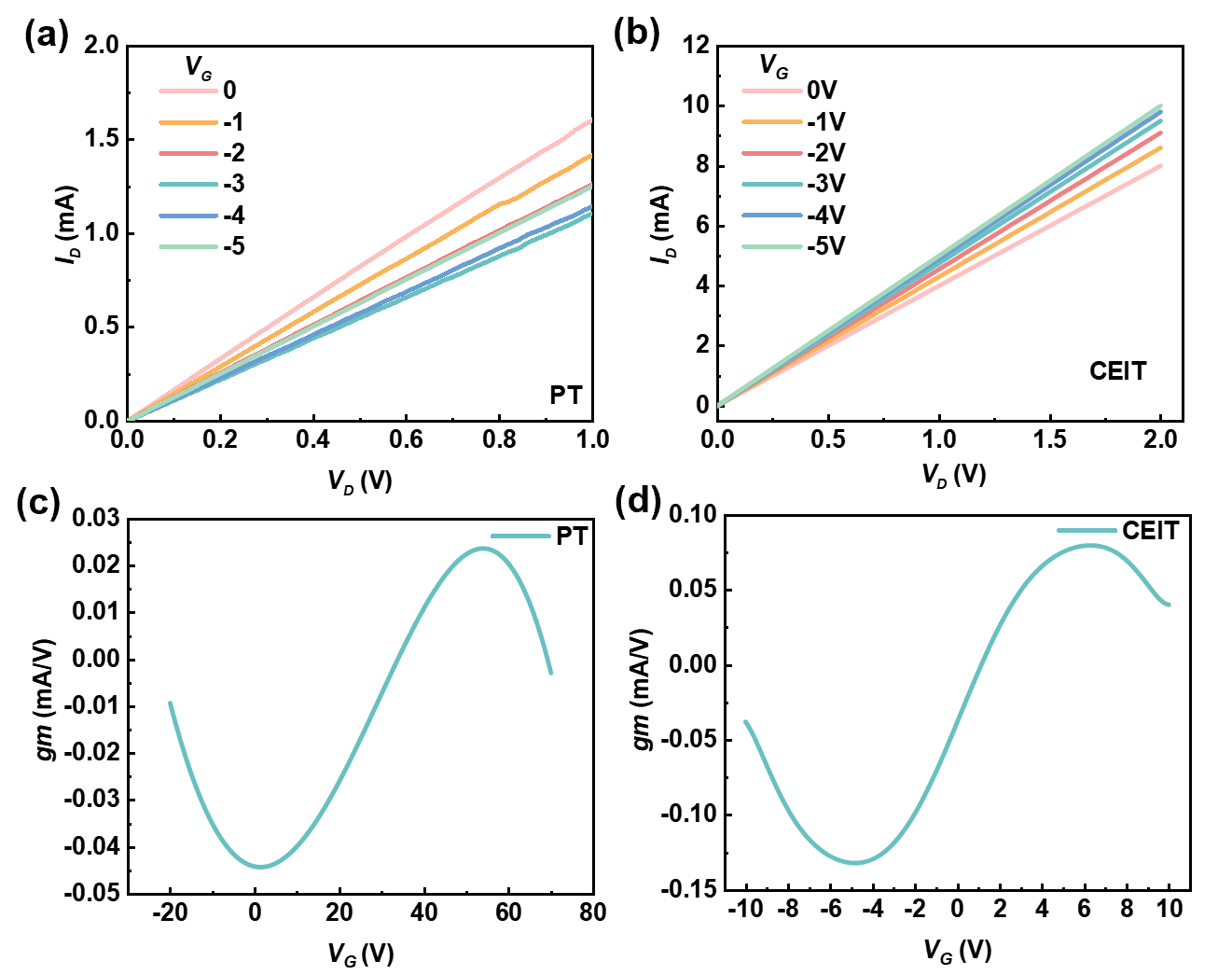


**Figure S14.** (a)(b) Output curves of graphene transferred by CEIT and PT method. (c)(d) Transconductance of graphene transferred by CEIT and PT methods.

**Figure S15.** (a-c) Transfer curves, output characteristics, and transconductance of the graphene sheet after the rinse with HCl solution, respectively. The scale bar in the figure is 25 µm.

**Figure S16.** In the beginning, a cold plate and electrode plates were placed in a 0°C chamber next to the freeze dryer. Copper-based graphene was fixed on the electrode plate. An external electric field of 0.8 V/µm was applied between two electrode plates. Before transferring, the freeze-dryer’s temperature was preset to -50°C. To avoid ice melting during the transfer process, the copper-based graphene with the electrode plates was rapidly transferred into the freeze dryer in a couple of seconds.

**Figure S17.** (a) Frozen of ice/graphene/cold plate; (b) Manual detachment process; (c) Successful detached. The detachment involved manually peeling at an angle of approximately 30-45°, applying suitable force to separate the ice from copper-based graphene. (d-f) The actual detaching process was conducted within a freeze-dryer.

**Figure S18.** (a) Patterning process of graphene; (b) SEM image of copper substrate graphene after patterning.

**Table S1.** Values of Potential Parameters.

| Particles | *σ_ij_* (Å) | *ε_ij_* (eV) | *Q* (e) | *θ* (º) |
| --- | --- | --- | --- | --- |
| O-O | 3.1660 | 0.0068 | -0.8476 |  |
| H-H | 0.0000 | 0.0000 | +0.4238 |  |
| O-H | 0.0000 | 0.0000 |  |  |
| O-Cu | 2.7530 | 0.0459 |  | 91.20 |
| H-Cu | 0.0000 | 0.0000 |  |  |
| C-Cu | 3.0023 | 0.0117 |  |  |
| O-C | 3.1900 | 0.0225 |  | 87.94 |
| H-C | 0.0000 | 0.0000 |  |  |
| O-Si | 3.0100 | 0.0245 |  |  |
| H-Si | 0.0000 | 0.0000 |  |  |

**Table S2.** Temperature parameters of freeze-drying process

| Step |  | Temperature (℃) | Time (h) | Pressure (kPa) | Notes |
| --- | --- | --- | --- | --- | --- |
| 1 |  | -50 | 50 | 101 | Transfer operation |
| 2 |  | -50 | 2 | ≤0.0001 | Freeze Drying |
| 3 |  | -40 | 2 | ≤0.0001 | Freeze Drying |
| 4 |  | -30 | 6 | ≤0.0001 | Freeze Drying |
| 5 |  | -20 | 1 | ≤0.0001 | Freeze Drying |
| 6 |  | -10 | 1 | ≤0.0001 | Freeze Drying |
| 7 |  | 0 | 1 | ≤0.0001 | Freeze Drying |
| 8 |  | 10 | 1 | ≤0.0001 | Freeze Drying |
| 9 |  | 25 | 1 | ≤0.0001 | Freeze Drying  Holding for 10 min |
